# Evaluating the Clinical Utility of Left Ventricular Strains in Severe AS: A Pilot Study with Feature-Tracking Cardiac Magnetic Resonance

**DOI:** 10.3390/biomedicines12092104

**Published:** 2024-09-14

**Authors:** Carmen Cionca, Alexandru Zlibut, Renata Agoston, Lucia Agoston-Coldea, Rares Ilie Orzan, Teodora Mocan

**Affiliations:** 1 Department of Internal Medicine, Iuliu Hatieganu University of Medicine and Pharmacy, 400012 Cluj-Napoca, Romania; 2 Department Physiology, Iuliu Hatieganu University of Medicine and Pharmacy, 400012 Cluj-Napoca, Romania; 3 Department of Internal Medicine, Emergency County Hospital, 400347 Cluj-Napoca, Romania; 4 Division of Cardiovascular Imaging, Department for Cardiology I, University Hospital Muenster, 48149 Muenster, Germany; 5 Department of Radiology, Affidea Hiperdia Diagnostic Imaging Center, 400012 Cluj-Napoca, Romania

**Keywords:** aortic stenosis, feature tracking cardiac magnetic resonance imaging, left ventricle global longitudinal strain, left ventricle global radial strain, left ventricle global circumferential strain

## Abstract

**Background:** Aortic valve stenosis (AS) is the most common degenerative valvular heart disease, significantly impacting the outcome. Current guidelines recommend valve replacement only for symptomatic patients, but advanced cardiovascular imaging, particularly cardiac magnetic resonance (CMR), may refine these recommendations. Feature-tracking CMR (FT-CMR) effectively assesses left ventricular (LV) strain and shows promise in predicting major adverse cardiovascular events (MACEs), though data on AS are limited. This study explored the role of CMR-derived LV strain in predicting MACEs occurrence in patients with severe AS. **Method:** We prospectively assessed 84 patients with severe AS and 84 matched controls. Global longitudinal (GLS), circumferential (GCS), and radial strain (GRS) were evaluated using FT-CMR. A composite endpoint—cardiac death, ventricular tachyarrhythmias, and heart failure hospitalization—was analyzed over a median follow-up of 31 months. **Results:** GLS was considerably reduced in AS patients (−15.8% vs. −19.7%, *p* < 0.001) and significantly predicted MACEs (HR = 1.24, *p* = 0.002) after adjusting for LVEF, 6 min walk distance, native T1, and late gadolinium enhancement. This underscores GLS’s unique and robust predictive capability for MACEs in severe AS patients. Kaplan–Meier curves and ROC analysis both showed that GLS had the highest predictive performance for MACEs, with an AUC of 0.857. **Conclusions**: GLS provided independent incremental predictive value for outcome.

## 1. Introduction

Aortic stenosis (AS) is the most common degenerative heart valve disease, significantly affecting morbidity and mortality [1]. Advanced imaging techniques enhance risk assessment and may refine aortic valve (AV) replacement criteria, even for asymptomatic patients. Cardiac magnetic resonance imaging (CMR) effectively quantifies AS using techniques like direct planimetry, the Gorlin method, and phase-contrast imaging [2]. CMR is capable of detecting myocardial fibrosis, a condition that greatly influences outcomes in AS patients [3,4].

Left ventricular (LV) strains, initially assessed using echocardiography, provide crucial insights into subclinical cardiovascular conditions and prognosis across various diseases [5]. Recently, global longitudinal strain (GLS) has gained prominence in managing AS. It detects early myocardial dysfunction with greater sensitivity than left ventricular ejection fraction (LVEF) [6] offering a refined approach to risk assessment and decision-making for severe, even asymptomatic, AS. Furthermore, GLS has proven effective in predicting outcomes post-transcatheter AV replacement (TAVR), surpassing LVEF in guiding clinical management [7]. Moreover, GLS has been proved to be an independent predictor of mortality in patients with AS, regardless of therapy approach [8].

CMR is a valuable tool for evaluating myocardial strain and aligns well with speckle-tracking echocardiography [9]. Feature-tracking CMR (FT-CMR) accurately measures 2D and 3D LV strains, including GLS, global radial (GRS), and global circumferential strain (GCS) [10,11]. GLS, GRS, and GCS from FT-CMR have reliably predicted post-myocardial infarction LV remodeling [9]. A meta-analysis showed that GLS predicts mortality in coronary artery disease and dilated cardiomyopathy patients [12], and it also forecasts cardiac mortality, heart transplantation, ventricular tachycardia, and major cardiovascular adverse events (MACEs) in myocarditis [13,14].

While echocardiography-based studies have started to explore the role of LV strains for diagnosing, risk stratifying, and predicting prognosis in AS, there is a notable gap in the literature regarding CMR-derived LV strain metrics. CMR’s advanced imaging capabilities offer potential for more detailed insights into LV remodeling and prognosis in AS, underscoring the need for further research.

The aim of this study was to assess the effectiveness of LV strain in risk stratification and prognosis prediction for patients with severe AS using CMR.

## 2. Materials and Methods

### 2.1. Study Population

This prospective study, conducted from March 2018 to May 2023, included patients with severe AS and an equal number of healthy volunteers, who were of similar age and gender to the AS group, but had no clinical evidence of cardiovascular disease. Evaluations were performed at the 2nd Department of Internal Medicine, Iuliu Hațieganu University of Medicine and Pharmacy, Cluj-Napoca, Romania, with CMR imaging done at Affidea Hiperdia Diagnostic Imaging Center, Cluj-Napoca [15,16].

**Figure 1:**
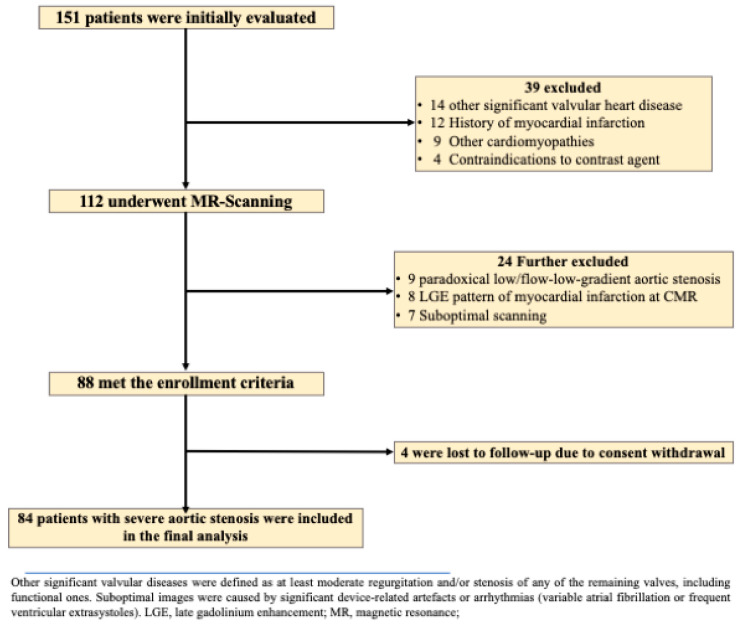
Flow-chart of the study design.

Eligibility for severe AS was confirmed by transthoracic echocardiography (using a General Electric, Vivid E95 Vingmed Ultrasound Horten Norway) conducted by a certified cardiologist, based on criteria such as peak aortic jet velocity ≥ 4 m/s, mean transvalvular gradient > 40 mmHg, or AV area (AVA) ≤ 1.0 cm^2^ [1]. The exclusion criteria are thoroughly reported in the flow-chart of the study (Figure 1). Patients with low-flow/low-gradient aortic stenosis who finally proved to be paradoxical were also excluded.

Both AS patients and healthy volunteers underwent a consistent protocol, including medical history, cardiac assessment, 6 min walk test (6MWD), ECG, 24 h Holter monitoring, biochemical analysis, echocardiography, and CMR imaging. Healthy volunteers were matched for age and sex but had no clinical evidence of cardiovascular disease. Regarding the AS subjects, 65 of them underwent open-heart surgery, while 19 TAVR.

The study was approved by the Ethics Committee of Iuliu Hațieganu University of Medicine and Pharmacy (decision number 196/10.03.2016) and adhered to the Declaration of Helsinki. All participants provided written consent before inclusion.

### 2.2. Image Acquisition and Analysis of Standard CMR Metrics

CMR imaging was performed with a Siemens 1.5 T Open Bore scanner (Magnetom Altea, Siemens Medical Solutions, Erlangen, Germany) following standard international protocols. Steady-state free precession (SSFP) sequences were utilized to evaluate biventricular function and mass in both short-axis and long-axis planes, covering the ventricles from base to apex [17]. Scanning parameters included a repetition time (TR) of 3.6 ms, echo time (TE) of 1.8 ms, flip angle of 60°, slice thickness of 6 mm, field of view of 360 mm, image matrix of 192 × 192 pixels, voxel size of 1.9 × 1.9 × 6 mm, and a temporal resolution of 25–40 ms reconstructed to 30 cardiac phases.

Late gadolinium enhancement (LGE) imaging was conducted 10 min post intravenous administration of 0.2 mmol/kg gadoteric acid (Clariscan, GE HealthCare AS, Oslo, Norway) using a segmented inversion-recovery gradient-echo sequence (TR 4.8 ms, TE 1.3 ms, inversion time 200–300 ms).

T1 mapping was performed with a modified Look-Locker sequence, employing 8 mm sections with a 2 mm gap, a field of view of 340 mm, and a matrix of 192 for a mid-ventricular short-axis slice. Native T1 times were measured before and after contrast infusion, with normal values considered to be 950 ± 21 ms [18]. Extracellular volume (ECV) was computed using the formula ECV = (1 − hematocrit) × [(1/pT1m^−1^/nT1m)/(1/pT1b^−1^/nT1b)], with reference values of 25 ± 4% [18].

Two experienced observers—a cardiologist and a radiologist, each with over 15 years of CMR experience and blinded to clinical data—evaluated all images. Measurements of LV end-diastolic volume (LVEDV), end-systolic volume (LVESV), LVEF, and LV end-diastolic mass (LVM) were derived from short-axis cine-SSFP images. Epicardial and endocardial borders were traced semi-automatically at end-diastole and end-systole using Syngo Virtual Cockpit Software 3 VB20A_HF08. All volumes were indexed to body surface area. The presence, distribution, and mass of LGE were evaluated from short-axis images using a 17-segment model, with a threshold of 5 standard deviations above normal myocardial signal intensity. LGE extent was reported in grams and as a percentage of LVM [19].

### 2.3. Feature-Tracking CMR

**Figure 2:**
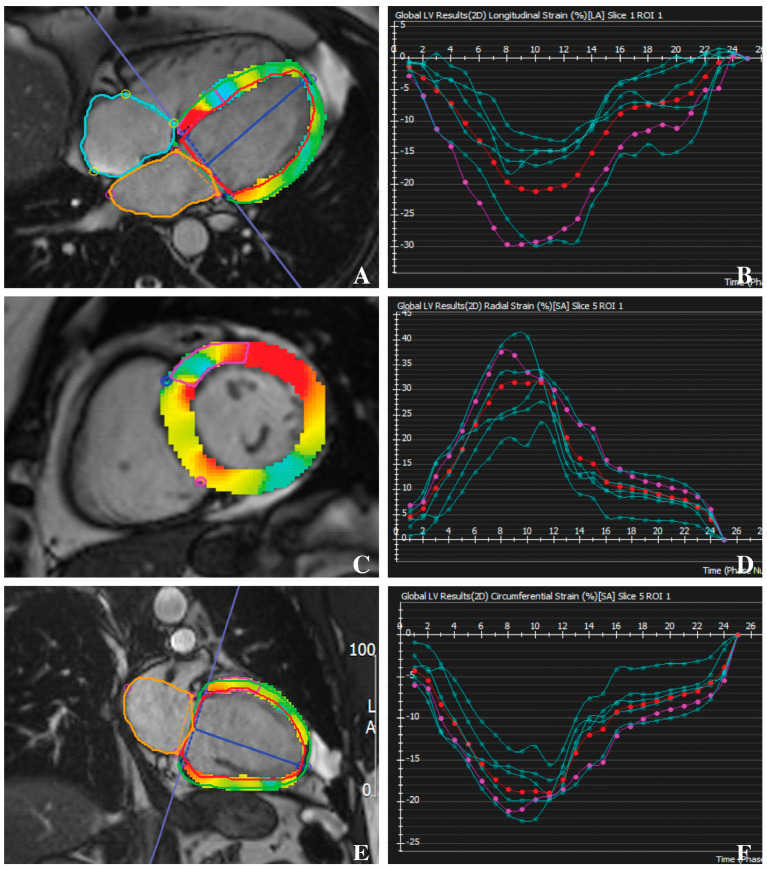
Example of post-processing feature-tracking technique using standard bSSF-CMR Images. The epicardial und endocardial contours are correspondingly drawn and LV strain metrics are graphically reported using the AHA 17-Segment model: GLS (**A**,**B**), GRS (**C**,**D**), and GCS (**E**,**F**). Usually, GLS and GCS show as negative percentage (**B**,**D**), whereas GRS show as positive one (**F**). Abbreviations: AHA, American Heart Association; GLS, global longitudinal strain; GCS, global circumferential strain; GRS, global radial strain; bSSF-CMR, balanced steady-state free precession cardiac magnetic resonance.

Post-processing of CMR images was conducted retrospectively using the cvi42 software package (version 5.16, Circle Cardiovascular Imaging Inc., Calgary, AB, Canada). GLS and GRS were evaluated from two-, three-, and four-chamber LV long-axis views, while GCS was assessed using basal, mid-ventricular, and apical LV short-axis views. Both endocardial and epicardial contours were manually delineated at end-diastole and end-systole, maintaining consistency across both long- and short-axis views for all strain parameters. During contouring, structures such as the LV outflow tract, trabeculae, papillary muscles, pericardium, and epicardial fat were excluded. Strain measurements were performed both regionally and globally, with LV segmentation following the American Heart Association 17-segment model, excluding segment 17 as previously described [17]. Patients with significantly inaccurate or improper images and contours were excluded from the analysis [20]. Segmental strain values were visualized using bulls-eye plots generated with the Python package Matplotlib (Figure 2).

### 2.4. Clinical Outcomes

Patients were followed up for a median of 31 months (interquartile range: 3 to 62 months) through a combination of hospital visits and telephone calls. The primary endpoint was a composite of MACEs, encompassing cardiac death, ventricular tachyarrhythmias, non-fatal myocardial infarction, third-degree atrioventricular block, and heart failure hospitalization. Non-cardiac hospitalizations were excluded from the analysis.

### 2.5. Statistical Analysis

Data were analyzed using MedCalc (Version 19.1.7). Normality was assessed with the Kolmogorov–Smirnov test. Results are presented as median, mean ± SD, or percentage. Pearson’s or Spearman’s correlations examined relationships between LV standard parameters, baseline characteristics, and LV strain metrics. Hazard ratios (HR) for event prediction were calculated using Cox regression, with significant univariate variables included in stepwise multivariate models for composite endpoints. Event-free survival was estimated using the Kaplan–Meier method, and statistical significance was assessed with the log-rank test. Retrospective power calculations and prospective sample size estimates accounted for type I and II errors.

## 3. Results

### 3.1. Baseline Characteristics

Patients with severe AS exhibited notable differences compared to matched controls across various clinical and diagnostic parameters (Table 1). A total of 29 of them had bicuspid AV. The AS group had a mean age of 66 years, compared to 65 years in the controls. They had a higher body mass index (30.1 kg/m^2^ vs. 28.5 kg/m^2^, *p* = 0.05) and were more likely to be smokers (39.3% vs. 25.0%, *p* < 0.01). The prevalence of hypertension and diabetes was also higher in the AS group (69.0% vs. 51.2%, *p* = 0.05; 41.7% vs. 26.2%, *p* = 0.05, respectively). Electrocardiogram analysis revealed a significantly greater incidence of left bundle branch block (16.6% vs. 3.5%, *p* < 0.001) and right bundle branch block (15.5% vs. 2.8%, *p* < 0.001) among AS patients. Atrial fibrillation of any category (paroxysmal, persistent or permanent) was observed in 9.5% of AS patients but was not present in the controls. Echocardiographic evaluations showed markedly higher peak aortic velocity (4.43 m/s vs. 1.34 m/s, *p* < 0.001), peak transaortic gradient (81.2 mmHg vs. 7.6 mmHg, *p* < 0.001), and mean transaortic gradient (52.4 mmHg vs. 3.7 mmHg, *p* < 0.001) in AS patients. The AVA index was significantly reduced in the AS group (0.52 cm^2^/m^2^ vs. 3.2 cm^2^/m^2^, *p* < 0.001). Biomarker analysis revealed markedly elevated NT-proBNP levels in AS patients (median 673 pg/mL vs. 210 pg/mL in controls, *p* < 0.001).

### 3.2. CMR Characteristics

CMR analysis highlighted notable differences between patients with AS and healthy controls (Table 1). AS patients had a significantly higher LVEDV (80.6 mL/m^2^ vs. 62.2 mL/m^2^, *p* < 0.001) and LVESV (34.3 mL/m^2^ vs. 21.2 mL/m^2^, *p* < 0.001), respectively. They also showed increased LVM indexed (95.5 g/m^2^ vs. 62.1 g/m^2^, *p* < 0.001) and reduced LVEF (59.0% vs. 65.9%, *p* < 0.001).

Strain measurements indicated decreased GLS in AS patients (−15.8% vs. −19.7%, *p* < 0.001) and GCS (−17.9% vs. −21.3%, *p* < 0.001). Conversely, GRS was higher in controls (38.8% vs. 28.9% in AS, *p* < 0.001).

Native T1 relaxation time was significantly prolonged in AS patients (1034 ms vs. 971 ms in controls, *p* < 0.001), and ECV was elevated (27.3% vs. 25.2%, *p* < 0.001). LGE was observed in 47.6% of AS patients, with a mean mass of 15.6 g (Table 1).

CMR measurements were repeatedly performed on the same set of images, acquired from all patients in the study group. Intra- and inter-observer reproducibility of LVEF, GLS, GCS, and GRS measurements, and the assessment of LGE by CMR were excellent. The kappa coefficients of agreement were 0.89 (inter-reader) and 0.92 (intra-reader) for the assessment of LGE, and 0.96 (inter-reader) and 0.99 (intra-reader) for GLS (Table 2).

### 3.3. Correlation between LV Strain and LV Functional Parameters

GLS shows a strong positive correlation with GCS (r = 0.885, *p* < 0.0001), reflecting their combined role in assessing myocardial deformation. It negatively correlates with GRS (r = −0.523, *p* < 0.0001), indicating that lower GLS is associated with decreased radial strain. GLS also positively correlates with LVM (r = 0.444, *p* < 0.0001), highlighting the relationship between myocardial mass and reduced longitudinal strain. A positive correlation with LGE (r = 0.418, *p* < 0.0001) further emphasizes that GLS reflects myocardial fibrosis. There is no significant correlation between GLS and AVA (r = 0.095, *p* = 0.3923), suggesting GLS is more related to myocardial changes rather than AVA.

GCS negatively correlates with GRS (r = −0.511, *p* < 0.0001), indicating that reduced circumferential strain aligns with lower radial strain. GCS also shows a positive correlation with LGE (r = 0.376, *p* < 0.0001) and LVM (r = 0.411, *p* < 0.0001), supporting its role in evaluating myocardial fibrosis and mass. GCS does not significantly correlate with AVA (r = 0.027, *p* = 0.8057).

GRS is inversely related to both GLS and GCS, showing that lower radial strain corresponds with decreased longitudinal and circumferential strains. GRS also negatively correlates with LGE (r = −0.508, *p* < 0.0001), indicating that increased myocardial fibrosis is associated with lower radial strain. GRS does not significantly correlate with LVM (r = 0.111, *p* = 0.3162) or AVA (r = 0.111, *p* = 0.3162), suggesting limited direct relation to these parameters.

### 3.4. ROC Analysis of GLS and CMR Parameters for Predicting MACEs

**Figure 3:**
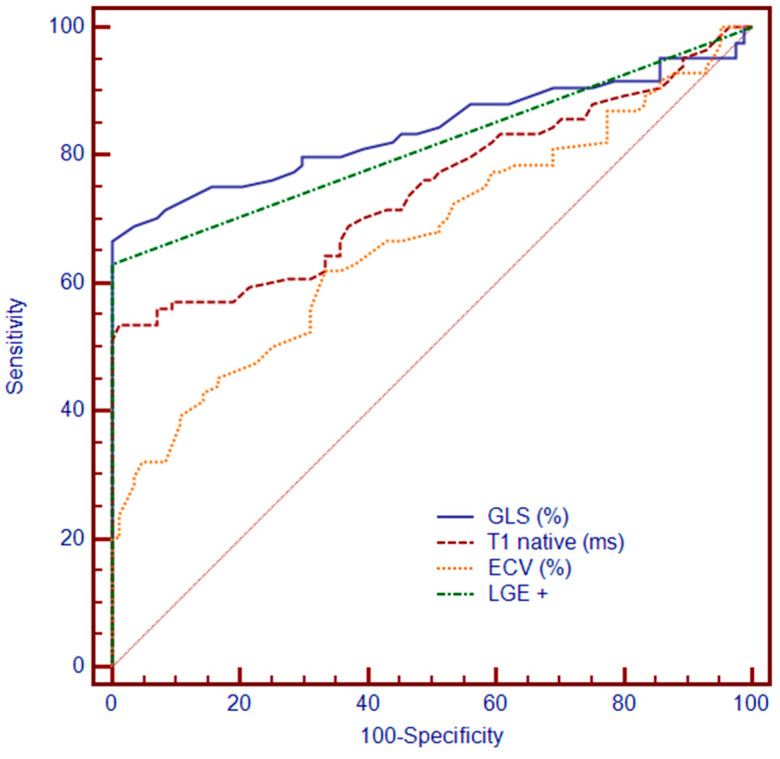
ROC analysis demonstrating the ability of GLS, T1 native, ECV, and LGE. Abbreviations: GLS, global longitudinal strain; LGE, late gadolinium enhancement; ECV, extracellular volume.

The incremental predictive ability of various parameters to forecast MACEs over a 31-month follow-up was calculated by receiver operating characteristic curve analysis for the combined end-point: GLS exhibited the highest predictive performance with an AUC of 0.839 (SE: 0.0336, 95% CI: 0.775 to 0.891), indicating a robust ability to predict MACEs. T1 native followed with an AUC of 0.749 (SE: 0.0388, 95% CI: 0.677 to 0.813), demonstrating moderate predictive value. ECV had an AUC of 0.670 (SE: 0.0419, 95% CI: 0.594 to 0.741), reflecting its less prominent but still significant role in predicting outcomes. LGE showed an AUC of 0.812 (SE: 0.0343, 95% CI: 0.745 to 0.868), indicating strong predictive capability, especially when combined with other measures (Figure 3).

### 3.5. Survival Analysis of GLS and CMR Parameters for Predicting MACEs

During a median follow-up period of 31 (3 to 62) months, 33 patients (39.3%) had MACEs: non-fatal myocardial infarction (n = 3), sustained ventricular arrhythmias (n = 3), third-degree atrioventricular block (n = 3), and hospitalization for heart failure (n = 24). Most patients (n = 23, 69.7%) with LGE on CMR imaging experienced MACEs during follow-up.

**Figure 4:**
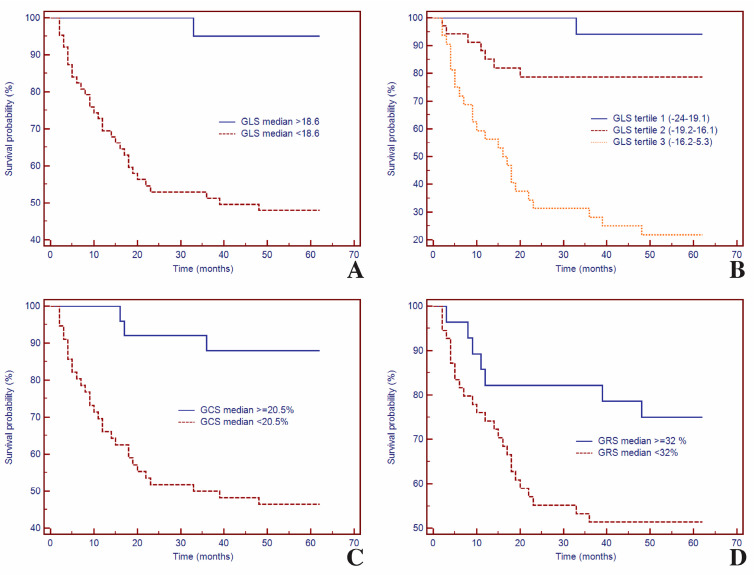
Kaplan–Meier curves for time-to-event analysis of GLS (**A**,**B**), GCS (**C**), and GRS (**D**). Abbreviations: GLS, global longitudinal strain; GCS, global circumferential strain; GRS, global radial strain.

Kaplan–Meier curves based on LV strain parameters, GLS, GCS, and GRS are presented in Figure 4. Kaplan–Meier curves for event-free survival showed a significantly higher rate of MACEs in patients with decreased GLS at CMR imaging (*p* < 0.001).

Thus, for specific thresholds, LV strain parameters significantly predicted MACEs: for GLS (HR = 14.27, 95% CI (6.82–29.88), *p* = 0.0005) (Figure 4A), for GLS tertile (HR = 34.41, 95% CI (21.17–53.87), *p* < 0.0001) (Figure 4B), for GCS (HR = 6.27, 95% CI (3.11–12.65), *p* = 0.0005) (Figure 4C), and for GRS (HR = 2.32, 95% CI (1.15–4.68), *p* = 0.0398) (Figure 4D).

### 3.6. Univariate and Multivariate Cox Analysis of LV Strain in Predicting MACEs

In the univariate analysis, several parameters were statistically significant for predicting MACEs (Table 3). These included BMI (HR 0.89, *p* = 0.01), diabetes mellitus (HR 1.02, *p* = 0.002), 6MWD (HR 0.99, *p* = 0.001), LVEDV index (HR 1.02, *p* = 0.003), and LVESV index (HR 1.04, *p* = 0.002). Additionally, LVEF (HR 0.94, *p* = 0.001), GLS (HR 1.21, *p* < 0.0001), and GCS (HR 1.20, *p* < 0.0001) were significant. Other significant parameters included GRS (HR 0.89, *p* = 0.005), native T1 (HR 1.03, *p* < 0.001), LGE mass index (HR 1.13, *p* < 0.001), and peak aortic velocity (HR 2.10, *p* = 0.033).

In the multivariate analysis, GLS (HR 1.19, 95% CI 1.08–1.44, *p* = 0.002) and LGE mass index (HR 0.97, 95% CI 0.88–1.55, *p* = 0.049) maintained their significance, highlighting their robust predictive ability for MACE. Other parameters such as 6MWD, LVEF, and native T1 did not retain significance in the multivariate model. This emphasizes the potential utility of GLS and LGE in clinical risk stratification for patients at risk of MACE. Thus, while multiple parameters were significant in univariate analysis, only GLS and LGE demonstrated robust predictive ability for MACE in the multivariate model, underscoring their potential utility in clinical risk stratification (*p* < 0.0001).

A stepwise Cox proportional-hazards models highlighted the significant role of GLS in predicting the composite outcome (Table 4).

### 3.7. Incremental Predictive Ability of LV Strain for Predicting MACEs

**Figure 5:**
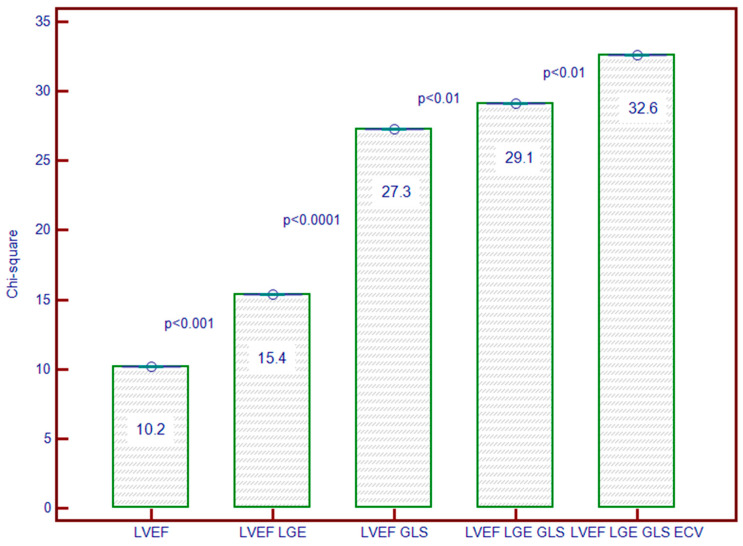
Incremental predictive value of GLS added to LVEF, LGE, and ECV. The *y*-axis represents the Chi-square values of the stepwise Cox proportional hazards models. Abbreviations: GLS, global longitudinal strain; LVEF, left ventricular ejection fraction; LGE, late gadolinium enhancement; ECV, extracellular volume.

Initially, LVEF alone demonstrated predictive value (Chi-square = 10.24; *p* = 0.0014). The addition of LGE to LVEF improved prediction (Chi-square = 15.46; *p* = 0.0032). However, the inclusion of GLS with LVEF and LGE markedly enhanced predictive accuracy (Chi-square = 27.04; *p* < 0.0001), underscoring GLS’s critical role. Further, incorporating ECV alongside LVEF, LGE, and GLS led to the highest predictive performance (Chi-square = 32.66; *p* < 0.0001), reinforcing the central importance of GLS in risk stratification (Figure 5).

## 4. Discussion

To our knowledge, this pilot study represents a pioneering investigation into the prognostic significance of LV strain metrics derived from CMR imaging in patients with severe AS. Characterized by narrowing of the AV and associated with significant cardiovascular risk, AS requires advanced predictive tools for accurate assessment of MACEs. While traditional CMR parameters such as LVEF and LGE have guided clinical decision-making, this research introduces CMR-based LV strain, particularly the GLS, as a potentially transformative metric.

CMR has become crucial for evaluating myocardial strain, utilizing various methods with distinct advantages and limitations. Tissue-tagging CMR, phase velocity mapping, fast cine displacement encoding with stimulated echo, strain-encoded imaging, and FT each offer unique benefits but also face challenges such as tag fading, low signal-to-noise ratio, and motion artifacts [21]. Weise Valdes et al. aimed to establish reference values for strain using FT and fast strain-encoded imaging, finding FT-GLS at −16.9 ± 1.8%, FT-GCS at −19.2 ± 2.1%, and FT-GRS at 34.2 ± 6.1%. Notably, fast strain-encoded GLS was significantly higher at −20.3 ± 1.8% (*p* < 0.001). Strain values were lower in men and correlated positively with cardiac muscle mass for FT-GLS and FT-GCS but negatively for FT-GRS. The study set clinical cut-off values and highlighted that this method generally yields higher GLS values compared to FT [22,23].

Recent studies highlight the prognostic significance of GLS measured by CMR, demonstrating its value beyond traditional metrics such as LVEF. In dilated cardiomyopathy, GLS was significantly lower in patients compared to healthy controls (−15.3% vs. −21.2%, *p* < 0.001) and provided additional risk stratification beyond LVEF [23]. For coronary artery disease patients with preserved LVEF, a GLS cutoff of −14.4% predicted adverse outcomes with an adjusted hazard ratio of 1.83 (95% CI: 1.28–2.61, *p* = 0.001), underscoring GLS’s sensitivity in risk prediction [24]. In acute myocarditis, GLS values ≤ −20% were linked to higher rates of MACEs, indicating its role in enhancing risk assessment even with normal LVEF [25]. Thus, GLS measured by CMR shows promise as a valuable prognostic tool in cardiac conditions. However, as research is still evolving, further studies are needed to fully establish its clinical utility and integrate it into routine practice.

The study by Santos et al. (2024) is the sole investigation to explore LV remodeling patterns in severe AS using CMR before and after surgical AV replacement. It aimed to assess the prevalence and characteristics of LV remodeling in 130 patients (mean age 71 years, 48% men) and how these change post-AVR. Preoperatively, LV hypertrophy was present in 49% of patients, with concentric hypertrophy being the most common pattern (44%). After AV replacement procedures, 60% of patients exhibited normal LV geometry, while 27% had concentric remodeling. Notably, asymmetric LV wall thickening, observed in 55% of patients before surgery, persisted in 35% postoperatively but shifted from basal to mid-septal regions. This study underscores the diverse LV remodeling patterns in AS and the significant changes following AVR, emphasizing CMR’s crucial role in monitoring LV adaptation [26].

Our study underscores that GLS is a robust and independent predictor of MACEs in patients with severe AS. We found GLS to be a superior prognostic tool compared to traditional parameters such as 6MWD, LVEF, native T1, and LGE mass index. GLS achieved an AUC of 0.857, outstripping the predictive performance of conventional CMR measures.

Furthermore, the main findings in the current research align with aforementioned research indicating that GLS offers significant incremental value over LVEF, LGE, and ECV in various cardiovascular conditions. Notably, our study is the first to confirm these insights specifically in the context of severe AS. Multivariate analysis showed that GLS remained a strong predictor of MACEs, with other parameters like LVEF and ECV losing predictive significance when adjusted for GLS. The hazard ratio for GLS was 2.26 (95% CI: 1.52–3.39, *p* < 0.001) for all-cause mortality, affirming its critical role in risk stratification. Therefore, patients with severe AS who appear to be asymptomatic might benefit from GLS assessment to refine the criteria for AV replacement procedures. However, further studies are still needed.

This pioneering research demonstrates that GLS enhances risk prediction in severe AS, corroborating findings from studies on other cardiovascular conditions. However, it also highlights the need for further validation to fully establish GLS’s role in clinical risk assessment for AS patients.

### Study Limitations

Nevertheless, several limitations must be acknowledged. This study was conducted at a single center with a relatively small sample size, which may limit the generalizability of the findings. The follow-up period of 31 months might be relatively short, potentially affecting the comprehensiveness and reliability of the outcome assessments. Furthermore, the study did not separately analyze patients with congenital AV diseases, such as bicuspid AVs, which could have distinct prognostic implications. Therefore, while the study provides valuable insights, further research is needed to validate these results in larger, more diverse cohorts and to assess long-term outcomes.

## 5. Conclusions

Our study is the first to establish CMR-based GLS as a robust predictor of MACEs in AS. GLS was strongly correlated with myocardial deformation and fibrosis, and it uniquely retains significant predictive value for MACEs in multivariate analysis. This underscores GLS’s potential to enhance clinical risk stratification for patients with AS.

## Figures and Tables

**Table 1:** Baseline characteristics of patients in the study.

Variables	AS All Patients n = 84	Controls n = 84	*p*-Value
**Clinical characteristics**			
- Age, mean (SD), years	66 (8.9)	65 (8.5)	NS
- Male gender, n (%)	46 (54.7)	48 (57.1)	NS
- Body mass index, kg/m^2^, mean (SD)	30.1 (4.9)	28.5 (4.2)	0.05
- 6MWD, m, mean (SD)	415 (138)	597 (102)	<0.001
- Smokers, n (%)	33 (39.3)	21 (25.0)	<0.01
- Hypertension, n (%)	58 (69.0)	41 (51.2)	0.05
- Diabetes mellitus, n (%)	35 (41.7)	22 (26.2)	0.05
**Medication**			
- Beta-blockers, n (%)	58 (69.0)	8 (9.5)	<0.001
- ACEIs or ARBs, n (%)	56 (66.6)	11 (13.1)	<0.001
- Calcium channel blockers, n (%)	24 (28.5)	6 (7.1)	<0.001
- Diuretics, n (%)	48 (57.1)	6 (7.1)	<0.001
- Anticoagulant, n (%)	17 (20.2)	-	NA
- Antiarrhythmic, n (%)	16 (19.0)	-	NA
**Electrocardiogram**			
- Atrial fibrillation, n (%)	8 (9.5)	-	NA
- Left bundle branch block, n (%)	14 (16.6)	3 (3.5)	<0.001
- Right bundle branch block, n (%)	13 (15.5)	2 (2.8)	<0.001
- Significant Q waves, n (%)	4 (4.7)	-	NA
**Echocardiography**			
- Peak aortic velocity, m/s, mean (SD)	4.43 (0.44)	1.34 (0.29)	<0.001
- Peak transaortic gradient, mmHg, mean (SD)	81.2 (17.1)	7.6 (2.23)	<0.001
- Mean transaortic gradient, mmHg, mean (SD)	52.4 (13.9)	3.7 (0.71)	<0.001
- AVA index, cm^2^/m^2^, mean (SD)	0.52 (0.08)	3.2 (0.06)	<0.001
**Biomarkers**			
- NT-proBNP, pg/mL, median (IQR)	673 (170–1960)	210 (60–330)	<0.001
**CMR parameters**			
- LVEDV index, mL/m^2^, mean (SD)	80.6 (20.2)	62.2 (14.8)	<0.001
- LVESV index, mL/m^2^, mean (SD)	34.3 (15.2)	21.2 (5.9)	<0.001
- LVM index, g/m^2^, mean (SD)	95.5 (23.5)	62.1 (14.7)	<0.001
- LVEF, %, mean (SD)	59.0 (9.3)	65.9 (4.7)	<0.001
- GLS, %, mean (SD)	−15.8 (3.9)	−19.7 (1.2)	<0.001
- GCS, %, mean (SD)	−17.9 (3.6)	−21.3 (1.4)	<0.001
- GRS, %, mean (SD)	28.9 (4.3)	38.8 (7.1)	<0.001
- T1 native, ms, mean (SD)	1034 (80.2)	971 (17.2)	<0.001
- ECV, % mean (SD)	27.3 (3.7)	25.2 (2.5)	<0.001
- LV-LGE, n (%)	40 (47.6)	-	NA
- LV-LGE mass, g/m^2^, mean (SD)	15.6 (6.2)	-	NA

Abbreviations: ACEI, angiotensin-converting enzyme inhibitor; ARB, angiotensin receptor blocker; AVA, aortic valve area; ECV, extracellular volume; GCS, left ventricular circumferential strain; GLS, left ventricular global longitudinal strain; GRS, left ventricular radial strain; LV-LGE, left ventricular late gadolinium enhancement; LVEDV, left ventricular end-diastolic volume; LVESV, left ventricular end-systolic volume; LVM, left ventricular mass; LVEF, left ventricular ejection fraction; 6MWD, six-minute walk distance; NT-proBNP, N-terminal pro-brain natriuretic peptide; n, number of patients; NA, Not Applicable; SD, standard deviation; IQR, interquartile range.

**Table 2:** Reproducibility inter- and intra-reader agreement of CMR measurements.

Parameters	Coefficient Kappa 95%	Confidence Interval	Standard Error
Inter-reader			
- LVEF	0.94	0.908 to 0.970	0.028
- GLS	0.96	0.917 to 0.981	0.021
- GCS	0.92	0.897 to 0.956	0.036
- GRS	0.90	0.873 to 0.952	0.049
- LGE	0.89	0.801 to 0.934	0.074
Intra-reader			
- LVEF	0.99	0.982 to 0.991	0.003
- GLS	0.96	0.958 to 0.984	0.017
- GCS	0.93	0.913 to 0.967	0.029
- GRS	0.92	0.911 to 0.944	0.038
- LGE	0.91	0.908 to 9.943	0.035

Abbreviations: GCS, left ventricular circumferential strain; GLS, left ventricular global longitudinal strain; GRS, left ventricular radial strain; LGE, left ventricular late gadolinium enhancement; LVEF, left ventricular ejection fraction.

**Table 3:** Univariate and Multivariate Cox Analysis testing between studied parameters and MACEs.

	No Events n = 51	Events n = 33	Univariate Analysis		Multivariate Analysis	
			Unadjusted HR (95% CI)	*p* Value	Adjusted HR (95% CI)	*p* Value
Age, years	66 (10.2)	67 (6.5)	1.00 (0.96–1.04)	NS		
Male gender, n, %	24 (47.0)	22 (66.7)	0.51 (0.25–1.06)	NS		
Body-mass index, kg/m^2^	31.0 (4.8)	28.7 (4.9)	0.89 (0.82–0.97)	0.01		
Systolic blood pressure, mmHg	130 (10.6)	130 (15.0)	1.00 (0.98–1.02)	NS		
Smokers, n %	19 (37.2)	14 (42.4)	0.98 (0.96–1.01)	NS		
Hypertension, n %	33 (64.7)	25 (75.7)	0.97 (0.68–1.03)	NS		
Diabetes mellitus, n %	20 (39.2)	15 (45.4)	1.02 (1.01–1.05)	0.002	0.98 (0.87–1.02)	NS
NT-proBNP, pg/mL	706 (170–935)	1034 (234–1960)	1.01 (1.00–1.02)	NS		
6MWD, m	464 (134)	338 (106)	0.99 (0.99–1.00)	0.001	0.97 (0.95–0.99)	NS
LVEDV index, mL/m^2^	75.2 (20.2)	90.1 (16.3)	1.02 (1.01–1.04)	0.003		
LVESV index, mL/m^2^	29.6 (13.1)	41.5 (15.4)	1.04 (1.02–1.06)	0.002		
LVM index, g/m^2^	93.3 (16.9)	101.9 (18.3)	1.01 (0.99–1.03)	NS		
LVEF, %	61.5 (8.1)	55.1 (9.6)	0.94 (0.90–0.98)	0.001	0.99 (0.94–1.02)	NS
GLS, %	−17.6 (3.0)	−13.8 (3.1)	1.21 (1.12–1.13)	<0.0001	1.19 (1.07–1.53)	0.003
GCS, %	−19.5 (3.2)	−15.6 (2.9)	1.20 (1.11–1.30	<0.0001		
GRS, %	30.2 (3.1)	27.6 (5.2)	0.89 (0.82–0.96)	0.005		
T1 native, ms	1022 (87)	1103 (70)	1.03 (0.99–1.08)	<0.001	0.97 (0.96–1.01)	NS
ECV, %	27.2 (3.9)	27.4 (3.4)	1.02 (0.98–1.11)	<0.01		
LGE mass index, g/m^2^	13.8 (2.3)	17.3 (5.6)	1.13 (1.01–1.42)	<0.001	0.97 (0.88–1.55)	0.049
Peak aortic velocity, m/s	4.34 (0.34)	4.55 (0.54)	2.10 (1.12–3.9)	0.033		
Peak aortic gradient, mmHg	78.8 (13.5)	84.8 (21.1)	1.04 (0.98–1.11)	NS		
Mean aortic gradient, mmHg	51.9 (12.9)	53.2 (15.6)	1.00 (0.98–1.02)	NS		
AVA index, cm^2^/m^2^	0.51 (0.08)	0.51 (0.08)	1.48 (0.29–7.37)	NS		

Abbreviations: AVA, aortic valve area; ECV, extracellular volume; GCS, left ventricular circumferential strain; GLS, left ventricular global longitudinal strain; GRS, left ventricular radial strain; LGE, late gadolinium enhancement; LVEDV, left ventricular end-diastolic volume; LVESV, left ventricular end-systolic volume; LVEF, left ventricular ejection fraction; LVM, left ventricular mass; 6MWD, six-minute walk distance n, number of patients; NT-proBNP, N-terminal pro-brain natriuretic peptide; NS, not significant.

**Table 4:** Stepwise multivariate proportional hazards model for the combined endpoint.

Variables	Model 1	Model 2	Model 3	Model 4	Model 5	Model 6
	HR 95%	HR 95%	HR 95%	HR 95%	HR 95%	HR 95%
Age	1.01 (0.96–1.39)	0.95 (0.91–0.99)	1.01 (0.97–1.05)	1.00 (0.96–1.04)	0.99 (0.95–1.03)	1.01 (0.97–1.05)
6MWD		0.99 (0.98–0.99) *				
LVEF			0.94 (0.90–0.96)			
ECV				1.02 (0.93–1.12)		
LGE					1.02 (1.01–1.36) **	
GLS						1.22 (1.12–1.32) *

Abbreviations: ECV, extracellular volume; GLS, left ventricular global longitudinal strain; LGE, left ventricular late gadolinium enhancement; LVEF, left ventricular ejection fraction; 6MWD, six-minute walk distance. * *p* < 0.0001; ** *p* < 0.01.

## Data Availability

Data are contained within the article.
